# TGF-β1-dependent expression of FOXS1 attenuates adipogenic potential and enhances a myofibroblast cellular phenotype

**DOI:** 10.1016/j.jbc.2025.110563

**Published:** 2025-08-05

**Authors:** Alexander H. Tavares, Scott P. Connelly, Daryn Maksat, Jane Zheng, Nabil Rabhi, Matthew D. Layne

**Affiliations:** Department of Biochemistry and Cell Biology, Boston University Chobanian & Avedisian School of Medicine, Boston, Massachusetts, USA

**Keywords:** forkhead box S1, transforming growth factor-β, myofibroblast, adipocyte stem cells, white adipose tissue, adipogenesis, fibrosis, fibroblast

## Abstract

White adipose tissue (WAT) fibrosis is a major determinant of obesity-induced cardiometabolic dysfunction and is characterized by excessive extracellular matrix deposition and myofibroblast activation. Transforming growth factor (TGF)-β1 is a profibrotic cytokine that potently induces myofibroblast activation in adipocyte stem cells (ASCs). How TGF-β1 orchestrates ASC activation in WAT fibrosis is not completely understood. We identified FOXS1, a member of the forkhead box transcription factor superfamily, as a transcriptional target of TGF-β1 signaling in primary human WAT ASC (hASC). FOXS1 potentiated TGF-β1-dependent upregulation of several myofibroblast genes (*e.g. Acta2*, *Col1a1*, *Fn1*, and *Il11*) in 10T1/2 fibroblasts. FOXS1 also attenuated the induction of several adipogenic factors (*e.g. Pparg*, *Stat5a*, *Fabp4*, and *Adipoq*) in 10T1/2 fibroblasts and sensitized these cells to the antiadipogenic effects of TGF-β1. Furthermore, loss of endogenous FOXS1 improved adipogenic permissiveness and activated proadipogenic gene programs in 10T1/2 cells, even after TGF-β1 stimulation. These results indicate that FOXS1 is a positive regulator of profibrotic TGF-β1-dependent cellular responses, orchestrating the regulation of molecular phenotypes that promote myofibroblast activation and block adipogenesis. These findings offer novel insight into the TGF-β1-dependent roles of FOXS1 in fibroblasts within the context of profibrotic ASC activation and provide a foundation for further investigation into the role of FOXS1 in WAT fibrosis and obesity-induced cardiometabolic dysfunction.

In obesity, white adipose tissue (WAT) fibrosis significantly contributes to WAT dysfunction and cardiometabolic disorder (1, 2, 3). WAT expands through *de novo* adipogenesis (hyperplasia), where new adipocytes arise from tissue-resident adipocyte stem cells (ASCs) (2). During chronic over nutrition, WAT expansion shifts to hypertrophy, where preexisting adipocytes increase in size to store excessive dietary lipid (2, 4). WAT hypertrophy is associated with hypoxia, adipocyte necrosis, and chronic inflammation, which together, contribute to progressive WAT fibrosis (5). Myofibroblast activation and extracellular matrix (ECM) accumulation are hallmarks of obesity-induced WAT fibrosis (6). The association between excessive WAT ECM and poor clinical outcomes in obese individuals is well established (3, 7, 8). To that end, targeting WAT fibrosis remains an enticing avenue for novel therapeutics for obesity-induced cardiometabolic dysfunction.

Multipotent fibroblasts are critical for maintaining tissue ECM and specialized cell populations (9). WAT ASC are comprised of fibroblasts and mural cells, however, lineage tracing has mapped most new adipocytes to platelet derived growth factor receptor (PDGFR⍺^+^) fibroblasts (10). Fibroblast-to-myofibroblast activation is also a key driver of tissue fibrosis (9, 11). Myofibroblasts are characterized by increased ECM production and a contractile phenotype, marked by alpha-smooth muscle actin (⍺-SMA/*ACTA2*) expression (9, 11). PDGFR⍺^+^ ASC isolated from obese WAT have marked expression of ECM proteins (type I collagen, fibronectin) and ⍺-SMA (12). Expression of many activated myofibroblast factors (*e.g.* ⍺-SMA) blocks adipogenesis, further shifting dysfunctional WAT expansion toward hypertrophy (13).

During fibrotic WAT remodeling, ASC respond to a milieu of profibrotic stimuli. Transforming growth factor (TGF)-β1 is a potent profibrotic cytokine that induces fibrotic tissue remodeling and myofibroblast activation (14). Canonical TGF-β1 signaling occurs through dimerization of TGFβ receptor (TβR) II with TβRI (ALK5), which phosphorylates SMAD2/3 (14). Phosphorylated SMAD2/3 forms a complex with SMAD4 and translocates to the nucleus to regulate TGF-β1-dependent gene transcription (14). Genes regulated by the SMAD2/3 axis of the TGF-β1 signaling cascade are closely associated with tissue remodeling and fibrosis (15, 16).

In obesity, TGF-β1 is upregulated in WAT depots, while circulating levels of TGF-β1 increase with body mass index (17). Furthermore, ablating macrophage-derived TGF-β1 or TGF-β1 signaling in adipose tissue protects mice from obesity-induced WAT fibrosis and metabolic dysfunction (18, 19). TGF-β1 also attenuates fibroblast adipogenesis by inhibiting the transcriptional activity of CCAAT-enhancer-binding protein (C/EBP)β and C/EBPδ, which coordinate the induction of C/EBP⍺ and peroxisome proliferator-activated receptor gamma (PPARγ), the central adipogenic transcriptional regulators (20, 21). Elucidating the full scope of TGF-β1-dependent cellular responses in WAT ASC could illuminate new therapeutic targets for promoting WAT hyperplasia and attenuating myofibroblast activation.

The aim of this study was to characterize global transcriptomic changes to acute and chronic TGF-β1 signaling in WAT ASC and identify novel downstream effectors that may regulate profibrotic ASC myofibroblast activation and block ASC adipogenesis. In primary human WAT ASC (hASC), we identified FOXS1, a member of the forkhead box (FOX) transcription factor superfamily, as a TGF-β1 gene target induced after acute and chronic TGF-β1 signaling. Follow-up studies in multipotent 10T1/2 fibroblasts highlighted several molecular phenotypes associated with augmented FOXS1 expression related to the induction of myofibroblast differentiation and early and late adipogenesis. The results of this study illuminate FOXS1-dependent cellular responses to TGF-β1 associated with myofibroblast activation and adipogenic permissiveness.

## Results

### Global transcriptional responses to acute and chronic TGF-β1 stimulation in primary omental hASC

**Figure 1:**
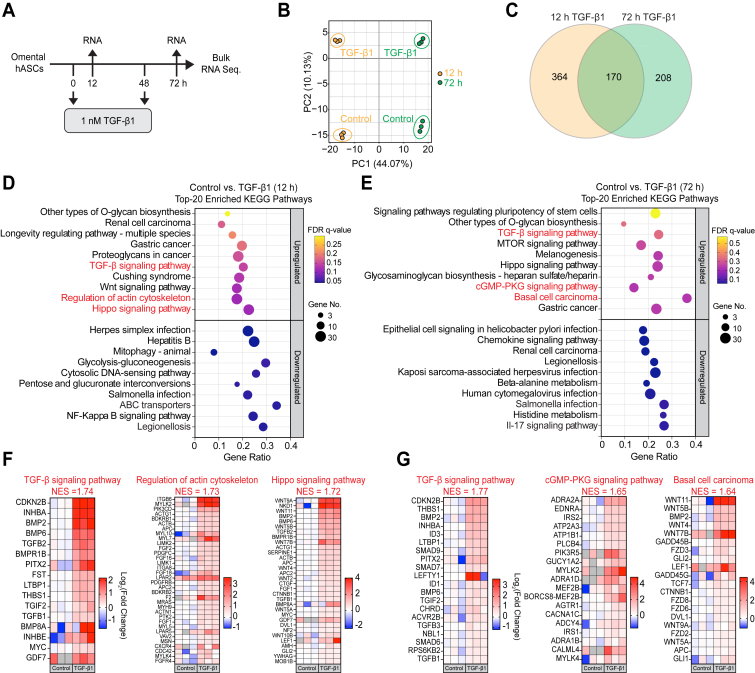
**hASC transcriptional responses to acute and chronic TGF-β1.***A*, scheme of TGF–β1 stimulation in hASC for bulk RNA sequencing. Cells were serum-starved overnight then stimulated with 1 nM TGF-β1 for 12 h and 72 h. *B*, PCA analysis of 12 h and 72 h transcriptional responses in control and TGF-β1 stimulated hASC. *C*, Venn diagram of unique and shared DEGs across TGF-β1 time points relative to time-matched controls. *D* and *E*, GSEA of the top 20 upregulated and downregulated KEGG pathways in 12 h and 72 h TGF-β1 stimulated hASC. *G* and *F*, heatmaps of core gene enrichments from selected KEGG pathways in 12 h and 72 h TGF-β1 stimulated hASC. Gene expression is reported as log_2_ (fold change) relative to time-matched controls. DEG, differentially expressed gene; GSEA, gene set enrichment analysis; hASC, human WAT adipocyte stem cell; KEGG, Kyoto encyclopedia of genes and genomes; NES, normalized enrichment score; PCA, principal component analysis; TGF, transforming growth factor.

TGF-β1 is a well-established profibrotic mediator that promotes ASC myofibroblast activation and drives fibrotic WAT remodeling (14, 17). We sought to elucidate the global transcriptomic changes downstream of acute and chronic TGF-β1 signaling in WAT ASC to identify cellular factors that may promote ASC myofibroblast activation and suppress ASC adipogenesis. Primary hASC were stimulated with TGF-β1 for 12 h or 72 h and purified RNA was subjected to bulk RNA sequencing (Fig. 1*A*). Cells stimulated with TGF-β1 for 72 h received a second TGF-β1 stimulation 24 h prior to RNA collection to induce chronic TGF-β1 responses (Fig. 1*A*).

Principal component analysis revealed clear clustering of 12 h and 72 h control and TGF-β1 stimulated hASC, indicating distinct gene expression profiles across groups and time points (Fig. 1*B*). Notably, 12 h and 72 h TGF-β1 stimulated hASC clustered distinctly from each other (Fig. 1*B*). Differential gene expression analysis defined differentially expressed genes (DEGs) between time-matched control and TGF-β1 stimulated hASC. Across 12 h and 72 h TGF-β1 time points, 742 DEGs were identified, with 364 DEGs unique to 12 h TGF-β1, 208 DEGs unique to 72 h TGF-β1, and 170 DEGs common between both time points (Fig. 1*C*, Table S1).

Gene set enrichment analysis (GSEA) using the Kyoto encyclopedia of genes and genomes (KEGG) database revealed enrichment for several upregulated and downregulated (DN) pathways in 12 h and 72 h TGF-β1 stimulated hASC (Tables S2 and S3). Top pathways upregulated after 12 h TGF-β1 included the TGF-β signaling pathway, regulation of actin cytoskeleton, and the Hippo signaling pathway (Fig. 1*D*). Top pathways upregulated after 72 h TGF-β1 included the TGF-β signaling pathway, the cGMP-PKG signaling pathway, and basal cell carcinoma (Fig. 1*E*). As anticipated, KEGG TGF-β signaling was among the top upregulated pathways at 12 h and 72 h time points, which shared several enriched genes (*CDK2B*, *THBS1*, *BMP2*, *INHBA*, *LTBP1*, *PITX2, BMP6*, *TGIF2*, and *TGFB*). Enriched TGF-β signaling pathway genes unique to 12 h included *TGFB2*, *BMPR1B*, *FST*, *BMP8A*, *INHBE*, *MYC*, and *GDF7* (Fig. 1*F*). Enriched TGF-β signaling pathway genes unique to 72 h included *ID3*, *SMAD9*, *SMAD7*, *LEFTY1*, *ID1*, *CHRD*, *ACVR2B*, *TGFB3*, *NBL1*, *SMAD6*, and *RPS6KB2* (Fig. 1*G*).

GSEA enrichment of KEGG Hippo signaling (Fig. 1*G*) and KEGG basal cell carcinoma (Fig. 1*F*) was driven by a strong enrichment for many genes related to wingless-related integration site (Wnt) signaling (*e.g. WNT9A*, *WNT11*, *WNT5B*, and *WNT4*) and bone morphogenetic protein (BMP) signaling (e.g. BMP2, BMP6, and BMPR1B) which have various profibrotic and antifibrotic functions in tissue fibrosis (24, 25, 26). Enrichment for the KEGG regulation of actin cytoskeleton gene set (Fig. 1*F*) was driven by several genes involved in cell adhesion (*e.g. ITGB6*, *ITGA8*, and *ACTN*), cell contraction (*e.g. MYLK2*, *MYL10*, and *ACTN1*) and stress fiber formation (*e.g. ACTG1*, *ACTB*, *LIMK1/2*, and *CDC42*), which are hallmark cellular processes in myofibroblast activation (9, 11).

Top pathways DN in hASC after 12 h TGF-β1 stimulation included ABC transporters and legionellosis (Fig. S1*A*). Top pathways DN in hASC after 72 h TGF-β1 stimulation included the IL-17 signaling pathway and *salmonella* infection (Fig. S1*B*). These pathways represent gene sets associated with proinflammatory phenotypes. Negative enrichment of the immune signature pathways was primarily driven by downregulation of several chemokines (*e.g. CXCL6*, *CXCL1*, *CXCL2*, *CXCL3*, and *CCL2*) and interleukins (*e.g. IL12B*, *IL1B*, *IL6*, *IL1A*, and *IL18*), highlighting the potent immunoregulatory functions of TGF-β1 in hASC.

In all, these results highlight distinct gene subsets that were regulated by TGF-β1 after acute or chronic stimulation. Given that these gene subsets were defined in primary hASC, these results provide a strong foundation for further studies investigating poorly defined TGF-β1-dependent genes and pathways involved in ASC myofibroblast activation. These results also illuminate established TGF-β1-dependent genes that have lesser studied roles in WAT fibrosis.

### FOXS1 is a downstream target of TGF-β1 in primary omental hASC

**Figure 2:**
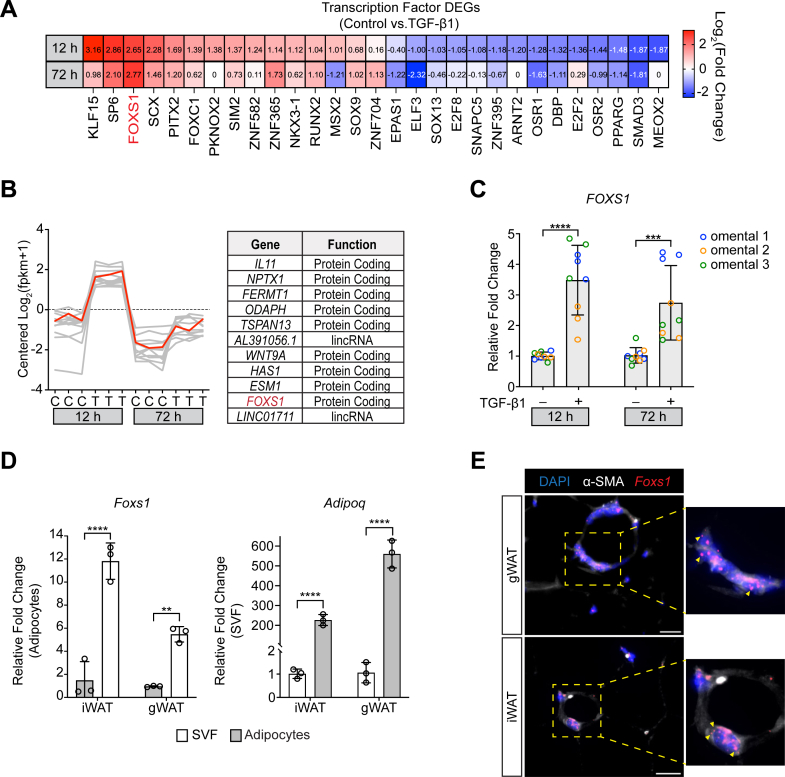
**FOXS1 is an acute and chronic TGF-β1 transcription factor target in hASC.***A*, heatmap of transcription factor DEGs across 12 h and 72 h TGF-β1 stimulated hASC. Gene expression is reported as log_2_ (fold change) relative to time-matched controls. *B*, cluster analysis of a subset of 11 DEGs that shared similar TGF-β1-dependent expression across 12 h and 72 h control (C) and TGF-β1 (T) time points. *C*, RT-qPCR of TGF-β1-dependent regulation of *FOXS1* in WAT hASC. Three independent WAT hASC isolates were serum-starved and stimulated with TGF-β1 as in (Fig. 1*A*). *D*, RT-qPCR of *Foxs1* and *Adipoq* in mouse iWAT and gWAT adipocytes and SVF. WAT from male mice (n = 3) was digested with 1 mg/ml type I collagenase at 37 °C and cellular fractions were separated by centrifugation. *Adipoq* was used as a fractionation control. *E*, RNAscope of *Foxs1* mRNA in mouse iWAT and gWAT tissue sections (n = 3). Representative sections were costained with ⍺-SMA and DAPI to visualize tissue vasculature and nuclei, respectively. RT-qPCR CT values were normalized to *18s* or *Ppia* expression. Two-way ANOVA with Bonferroni corrections for multiple comparisons (∗∗*p* < 0.01, ∗∗∗*p* < 0.001, ∗∗∗∗*p* < 0.0001). Data presented as mean ± S.D. The scale bars represent 25 μm. ⍺-SMA, alpha-smooth muscle actin; DAPI, 4′,6-diamidino-2-phenylindole; DEG, differentially expressed gene; FOXS1, forkhead box S1; gWAT, gonadal WAT; hASC, human WAT adipocyte stem cell; iWAT, inguinal WAT; RT-qPCR, real-time quantitative PCR; SVF, stromal-vascular fraction; TGF, transforming growth factor; WAT, white adipose tissue.

Many of the profibrotic cellular responses to TGF-β1 occur transcriptionally. We leveraged our RNA sequencing data in TGF-β1 stimulated hASC to elucidate changes in the expression of known transcription factors (TFs) that may be important for TGF-β1-dependent ASC myofibroblast activation. We identified 29 DEGs across 12 h and 72 h TGF-β1 time points that classified as a TF (Fig. 2*A*). Upregulated TF DEGs at both TGF-β1 time points included *SP6 (KLF14)*, *FOXS1*, *SCX*, *PITX2*, *ZNF365*, and *RUNX2.* DN TF DEGs at both TGF-β1 time points included *ELF3*, *OSR1*, *DBP*, *OSR2*, *PPARG*, and *SMAD3*. Interestingly, TFs that were differential DEGs between time points included *KLF15*, *FOXC1*, *PKNOX2*, *SIM2*, *ZNF582*, *NKX3.1*, *MSX2*, *SOX9*, *ZNF704*, *EPAS1*, *SOX13*, *E2F8*, *SNAPC5*, *ZNF395*, *ARNT2*, *E2F2*, and *MEOX2*.

Cluster analysis, which groups genes together based on their expression patterns, grouped *FOXS1* within a small cluster of 11 genes (nine protein coding, two lincRNAs) that had robust upregulation at 12 h and 72 h after TGF-β1 stimulation (Fig. 2*B*). Notably, many of the genes grouped in this cluster are established markers of myofibroblast activation (*e.g. IL11*, *WNT9A*, *HAS1*, and *ESM1*). We validated TGF-β1-dependent regulation of *FOXS1* in three independent primary omental hASC isolates by real-time quantitative PCR (RT-qPCR) (Fig. 2*C*). *FOXS1* was significantly upregulated in hASC isolates at 12 h and 72 h after TGF-β1 stimulation. Taken together, these results highlight *FOXS1* as a TGF-β1-responsive TF in primary hASC with a similar expression pattern to several genes with known roles in myofibroblasts, suggesting that FOXS1 may have important regulatory functions in profibrotic TGF-β1-dependent ASC activation.

### FOXS1 is enriched in the WAT stromal-vascular compartment

TGF-β1 responsive WAT ASC that contribute to fibrotic WAT remodeling reside within the stromal-vascular (SV) compartment (1, 2). To assess SV *FOXS1* expression and enrichment, subcutaneous inguinal WAT (iWAT) and visceral gonadal WAT (gWAT) was harvested from mice, digested, and separated into an stromal-vascular fraction (SVF) and adipocytes. RT-qPCR revealed a significant enrichment for *Foxs1* in the SVF compared to adipocytes in both WAT depots (Fig. 2*D*). Efficient WAT fractionation was confirmed by differential *Adipoq* expression (Fig. 2*D*). We used RNAscope to visualize *Foxs1* mRNA *in situ* within iWAT and gWAT depots. Tissues were costained with ⍺-SMA to mark the vascular regions where WAT ASCs reside. We observed clear *Foxs1* mRNA puncta within tissue areas marked by ⍺-SMA expression in both WAT depots, further indicating that FOXS1 is expressed by cells with the WAT SV compartment (Fig. 2*E*).

### FOXS1 expression is regulated in a SMAD2/3-dependent manner

**Figure 3:**
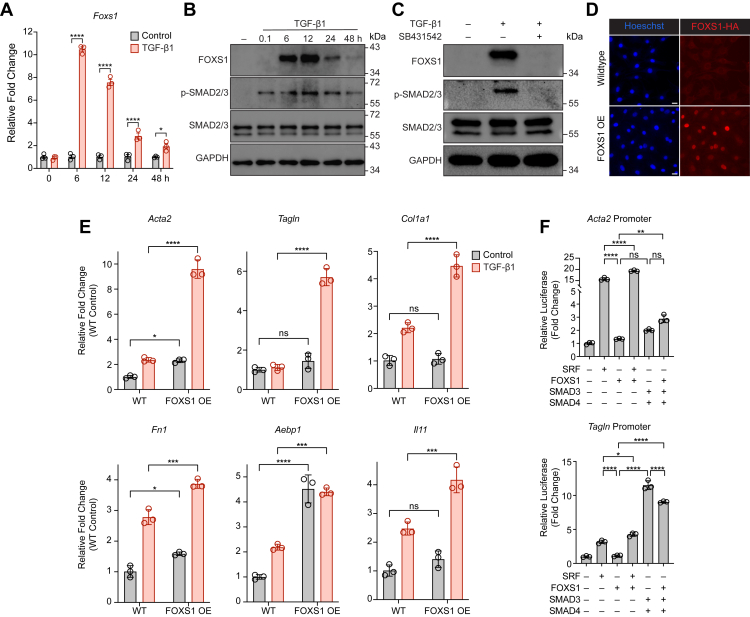
**FOXS1 potentiates profibrotic myofibroblast TGF-β1-dependent gene expression.***A*, kinetics of TGF-β1-dependent upregulation of *Foxs1* in 10T1/2 cells. Cells were serum-starved overnight and stimulated with 1 nM TGF-β1 for 6, 12, 24, and 48 h. *B*, kinetics of TGF-β1-dependent upregulation of FOXS1 protein expression in 10T1/2 cells. Cells were serum-starved and treated with TGF-β1 as in (*A*). Total protein (20 μg) was separated on 12% SDS-PAGE gels followed by western blot analysis using antibodies against FOXS1, phospho-SMAD2/3, total SMAD2/3, and GAPDH. *C*, inhibition of FOXS1 protein upregulation by blockade of SMAD2/3 phosphorylation in 10T1/2 cells. Cells were costimulated with 1 nM TGF-β1 and 5 μM of SB431542 (ALK5 inhibitor) for 6 h. Total protein (20 μg) was separated on a 12% SDS-PAGE gel followed by western blot analysis using antibodies against FOXS1, phospho-SMAD2/3, total SMAD2/3, and GAPDH. *D*, immunofluorescence of wildtype (WT) and FOXS1-HA overexpressed (FOXS1 OE) 10T1/2 cells. Cells were fixed in 4% PFA and stained with anti-HA primary and goat anti-rabbit Alexa Fluor 647 secondary antibodies. *E*, WT and FOXS1 OE 10T1/2 cells profibrotic transcriptional responses to TGF-β1. Cells were serum-starved and stimulated with 1 nM TGF-β1 for 24 h. Fold changes (2^−ΔΔCT^) were normalized to control WT 10T1/2 cells. *F*, activity of *Acta2* and *Tagln* promoters in 10T1/2 cells transfected with SRF, FOXS1, and SMAD3/4 alone or in combination. Promoter activity was accessed by normalized luciferase expression. Fold changes were calculated relative to empty vector luciferase values. RT-qPCR CT values were normalized to *18s* or *Ppia* expression. One-way or two-way ANOVA with Bonferroni corrections for multiple comparisons (ns = not significant, ∗*p* < 0.05, ∗∗∗*p* < 0.001, ∗∗∗∗*p* < 0.0001). Data presented as mean ± S.D. Data presented are representative from three independent experiments. The scale bars represent 25 μm. FOXS1, forkhead box S1; PFA, paraformaldehyde; RT-qPCR, real-time quantitative PCR; SMAD, suppressor of mothers against decapentaplegic; SRF, serum response factor; TGF, transforming growth factor.

10T1/2 cells are multipotent fibroblasts that respond to proadipogenic and profibrotic stimuli and are a useful tool for studying determinants of fibrotic and adipogenic phenotypes (27). We leveraged 10T1/2 cells to better understand the full kinetics of TGF-β1-dependent FOXS1 regulation. 10T1/2 cells were stimulated with TGF-β1 for 6, 12, 24, and 48 h. RT-qPCR revealed a significant upregulation of *Foxs1* that peaked 6 h after stimulation (Fig. 3*A*). In addition, FOXS1 protein expression was most abundant 12 h after TGF-β1 stimulation and rapidly turned over by 48 h. (Fig. 3*B*). Upregulation of the FOXS1 protein was preceded by increased phosphorylation of the canonical SMAD2/3 TGF-β1 effectors (Fig. 3*B*). Pharmacological inhibition (SB431542) of the TβRI (ALK5), which phosphorylates SMAD2/3, completely abrogated TGF-β1-dependent FOXS1 protein upregulation (Fig. 3*C*). These data indicate that FOXS1 is acutely regulated by TGF-β1 in a SMAD2/3-dependent manner. In many instances, genes that are regulated by the TGF-β-SMAD2/3 signaling axis attenuate the adipogenic capacity of WAT ASC and promote myofibroblast activation.

### FOXS1 potentiates profibrotic TGF-β1-dependent myofibroblast activation

To better understand the role of FOXS1 in cellular responses to TGF-β1, we generated 10T1/2 cells constitutively overexpressing HA-tagged FOXS1 (FOXS1 OE) (Fig. 3*D*). Wildtype (WT) or FOXS1 OE 10T1/2 cells were stimulated with TGF-β1 for 24 h. Relative to WT, FOXS1 OE 10T1/2 cells had significantly potentiated upregulation of several profibrotic genes that are established TGF-β1 targets essential for myofibroblast activation, including contractile cytoskeletal genes (*Acta2* and *Tagln*), ECM genes (*Col1a1*, *Fn1*, and *ACLP/Aebp1*), and profibrotic mediator genes (*Il11*) (Fig. 3*E*). We also assessed the regulation of ⍺-SMA protein expression by immunofluorescence in WT and FOXS1 OE 10T1/2 cells stimulated with TGF-β1 for 48 h (Fig. S2), which showed a significant increase in ⍺-SMA expression in the TGF-β1 stimulated FOXS1 OE cells relative to WT.

Many of the genes that were potentiated by FOXS1 are regulated downstream of TGF-β1 by SMAD3 or serum response factor (SRF) (28). To investigate if FOXS1 can transcriptionally cooperate with SMAD3 or SRF, 10T1/2 cells were transfected with luciferase reporter constructs for the *Acta2* or *Tagln* promoters. Cotransfection with FOXS1 alone did not markedly change *Acta2* or *Tagln* promoter activity relative to an empty vector control (Fig. 3*F*). Cotransfection of FOXS1 with SRF increased *Acta2* and *Tagln* promoter activity relative to SRF alone (Fig. 3*F*). Furthermore, cotransfection of FOXS1 with SMAD3/4 did not significantly change *Acta2* promoter activity and partially blunted *Tagln* promoter activity relative to SMAD3/4 alone. Taken together, these data indicate that FOXS1 potentiates the expression of TGF-β1-dependent profibrotic myofibroblast genes though potential cooperation with SRF transcriptional activity.

### FOXS1 mitigates adipogenesis potential and capacity

**Figure 4:**
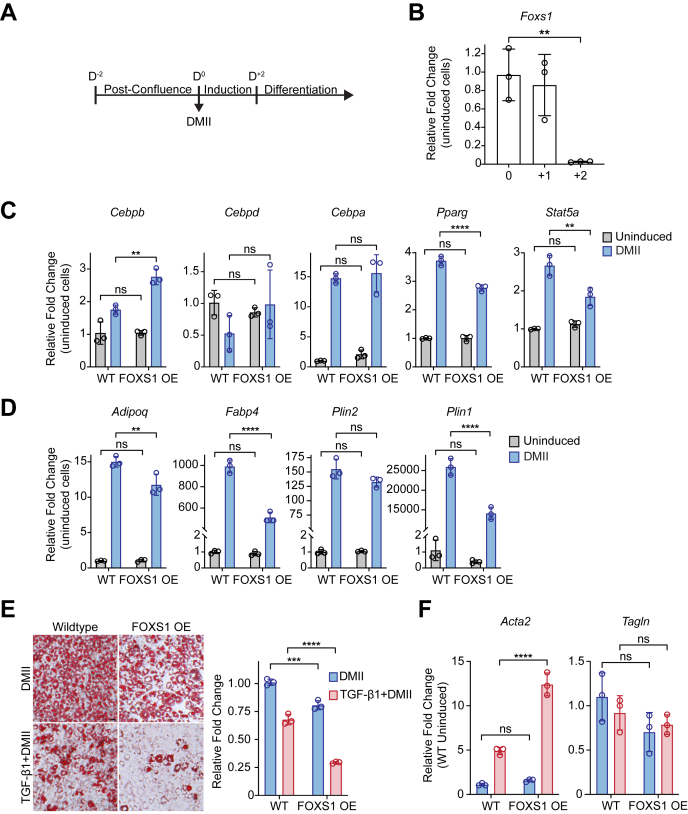
**FOXS1 is suppressive to induced adipogenic phenotypes.***A*, scheme of 10T1/2 adipogenic induction and differentiation. DMII induction media is supplemented with dexamethasone, three-isobutyl-1-methylxanthine, insulin, and indomethacin. *B*, *Foxs1* expression in 10T1/2 cells induced with DMII. Cells were harvested at D^0^, D^+1^, and D^+2^ after DMII. Fold changes (2^−ΔΔCT^) were normalized to uninduced 10T1/2 cells. *C*, regulation of early adipogenic transcription factor genes in WT and FOXS1 OE 10T1/2 cells induced with DMII for 48 h. Fold changes (2^−ΔΔCT^) were normalized to uninduced WT 10T1/2 cells. *D*, regulation of lipid metabolism and insulin sensitivity genes in WT and FOXS1 OE 10T1/2 cells induced with DMII for 48 h. Fold changes (2^−ΔΔCT^) were normalized to uninduced WT 10T1/2 cells. *E*, D^+6^ differentiation of WT and FOXS1 OE 10T1/2 cells. Cells that were treated with TGF-β1 were stimulated with 1 nM TGF-β1 for 8 h prior to DMII induction. Oil Red-O staining was quantified in 100% isopropanol and by measuring the absorbance at 500 nm. Absorbances were corrected to blank controls and fold changes are relative to DMII induced WT 10T1/2 cells. *F*, *Acta2* and *Tagln* expression in D^+6^ differentiated WT and FOXS1 OE 10T1/2 cells. Fold changes (2^−ΔΔCT^) were normalized to DMII induced WT 10T1/2 cells. RT-qPCR CT values were normalized to *18s* or *Ppia* expression. Two-way ANOVA with Bonferroni corrections for multiple comparisons (∗∗*p* < 0.01, ∗∗∗*p* < 0.001, ∗∗∗∗*p* < 0.0001). Data presented as mean ± S.D. Data presented are representative from three independent experiments. The scale bars represent 25 μm. DMII, dexamethasone, 3-isobutyl-1-methylxanthine, insulin, and indomethacin; FOXS1, forkhead box S1; RT-qPCR, real-time quantitative PCR; TGF, transforming growth factor.

Many cellular factors that promote myofibroblast phenotypes in WAT ASC also inhibit their adipogenic potential and ability to undergo terminal adipogenesis, further exacerbating tissue dysfunction in WAT fibrosis. Given that FOXS1 regulates a molecular profibrotic myofibroblast phenotype (Fig. 3*E*), we hypothesized that FOXS1 would repress the induction of adipogenic factors required for adipogenesis. 10T1/2 cells can be induced toward an adipogenic lineage using growth media supplemented with dexamethasone, 3-isobutyl-1-methylxanthine, insulin, and indomethacin (DMII) (Fig. 4*A*). During early adipogenesis, cellular factors that are repressive to adipogenic programming are often DN (29). We assessed *Foxs1* expression in 10T1/2 cells 0, 24, and 48 h after DMII stimulation (Fig. 4*B*). *Foxs1* was significantly DN 48 h after DMII. To determine if maintaining *Foxs1* expression during early adipogenesis augments proadipogenic gene expression, WT or FOXS1 OE 10T1/2 cells were stimulated with DMII for 48 h. Compared to WT, FOXS1 OE 10T1/2 cells had significantly mitigated upregulation of the adipogenic transcription factors *Pparg* and *Stat5a* (Fig. 4*C*). Induction of *Cebpa* and *Cebpd* was not significantly different, while Cebpb was significantly increased in FOXS1 OE 10T1/2 cells (Fig. 4*C*). FOXS1 OE 10T1/2 cells also had significantly mitigated upregulation of several adipogenic factors involved in lipid metabolism (*Plin1* and *Fabp4*) and insulin sensitivity (*Adipoq*) (Fig. 4*D*).

After DMII induction, 10T1/2 cells can be terminally differentiated into mature adipocytes, marked by their lipid uptake and storage capacity. WT and FOXS1 OE 10T1/2 cells were differentiated into mature adipocytes for 6 days (D6) after DMII induction and stained with Oil Red-O to visualize and quantify lipid accumulation. Compared to WT, FOXS1 OE 10T1/2 cells had significantly decreased lipid accumulation (Fig. 4*E*). Furthermore, WT and FOXS1 OE 10T1/2 cells were also stimulated with TGF-β1 for 8 h prior to DMII induction and D6 differentiation. As expected, TGF-β1 stimulation significantly blunted terminal adipogenesis in WT 10T1/2 cells (Fig. 4*E*). However, this effect was intensified in the FOXS1 OE 10T1/2 cells, which showed significantly less terminal adipogenesis after TGF-β1 stimulation compared to WT (Fig. 4*E*). Interestingly, TGF-β1 stimulated, D6-differentiated FOXS1 OE 10T1/2 cells had significantly greater *Acta2* expression compared to WT (Fig. 4*F*). These results indicate that FOXS1 blocks early adipogenic molecular phenotypes, which suppresses terminal adipogenesis. FOXS1 also sensitizes cells to the potent antiadipogenic properties of TGF-β1.

### Ablation of endogenous FOXS1 increases cellular adipogenic capacity and partial resistance to the antiadipogenic effects of TGF-β1

**Figure 5:**
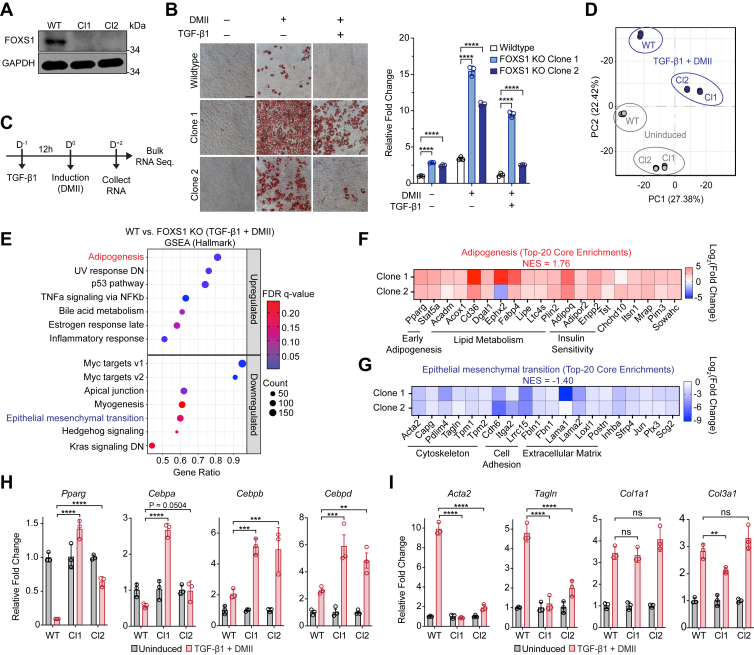
**FOXS1 ablation promotes proadipogenic molecular phenotypes and dampens myofibroblast molecular phenotypes.***A*, FOXS1 protein expression in WT 10T1/2 cells and two single-cell FOXS1 knockout (KO) 10T1/2 clones. Total protein (20 μg) was separated on a 12% SDS-PAGE gel followed by western blot analysis using antibodies against FOXS1 and GAPDH. *B*, D^+6^ differentiation of WT 10T1/2 cells and FOXS1 KO clones. Cells that were treated with TGF-β1 were stimulated with 1 nM TGF-β1 for 12 h prior to DMII induction. Oil Red-O staining was quantified in 100% isopropanol and by measuring the absorbance at 500 nm. Absorbances were corrected to blank controls and fold changes were calculated relative to undifferentiated WT 10T1/2 cells. *C*, scheme for TGF-β1 and DMII stimulation in WT or FOXS1 KO clones for global transcriptional analysis by bulk-RNA sequencing. *D*, PCA analysis for WT and FOXS1 KO clones in uninduced and TGF-β1+DMII stimulated conditions. *E*, GSEA of TGF-β1+DMII stimulated WT or FOXS1 KO clones using the mouse molecular signature hallmark gene sets. DEGs shared between FOXS1 KO clone 1 and 2 were used for GSEA. *F* and *G*, heatmaps of core gene enrichments in the upregulated adipogenesis (*F*) and downregulated epithelial-to-mesenchymal transition (*G*) hallmark pathway gene sets. *H* and *I*, RT-qPCR of early adipogenic transcription factor (*H*) or profibrotic myofibroblast (*I*) gene expression in WT or FOXS1 KO clones. Cells were stimulated as outlined in (*C*). Fold changes (2^−ΔΔCT^) were calculated relative to cell matched controls. All RT-qPCR CT values were normalized to *Ppia* expression. Two-way ANOVA with Bonferroni corrections for multiple comparisons (∗*p* < 0.05, ∗∗*p* < 0.01, ∗∗∗∗*p* < 0.0001). Data presented as mean ± S.D. Data presented are representative from three independent experiments. The scale bars represent 100 μm. DEG, differentially expressed gene; DMII, dexamethasone, 3-isobutyl-1-methylxanthine, insulin, and indomethacin; FOXS1, forkhead box S1; GSEA, gene set enrichment analysis; PCA, principal component analysis; RT-qPCR, real-time quantitative PCR; TGF, transforming growth factor.

To investigate how FOXS1 influences TGF-β1-dependent cellular responses within the context of antagonistic fibrotic and adipogenic stimuli, we generated FOXS1 knockout (KO) 10T1/2 cells using CRISPR-Cas9. Loss of endogenous FOXS1 expression was confirmed in two independent single-cell derived clones by western blot (Fig. 5*A*). We compared the potential of FOXS1 KO 10T1/2 cells to terminally differentiate into mature adipocytes relative to WT (Fig. 5*B*). Interestingly, FOXS1 KO clones exhibited significantly higher basal and induced lipid accumulation compared to WT (Fig. 5*B*). Furthermore, terminal adipogenesis in WT 10T1/2 cells was completely blocked after TGF-β1 stimulation, while FOXS1 KO clones were able to partially undergo adipogenesis after TGF-β1 stimulation. These results suggest that loss of FOXS1 increases cellular adipogenic potential and mitigates the potent antiadipogenic effects of TGF-β1.

### Ablation of endogenous FOXS1 promotes proadipogenic molecular phenotypes

Given that many of the cellular responses that coordinate adipogenesis occur within the first 48 h after DMII stimulation, we wanted to assess the global transcriptomic changes between WT and FOXS1 KO 10T1/2 cells during this early induction window after TGF-β1 stimulation. WT or FOXS1 KO 10T1/2 cells were stimulated with TGF-β1 for 12 h prior to stimulation with DMII for 48 h (Fig. 5*C*), after which, purified RNA was subjected to bulk RNA sequencing. Principal component analysis revealed distinct clustering of WT 10T/2 from FOXS1 KO clones 1 and 2 in uninduced and TGF-β1 + DMII stimulated conditions, indicating a close agreement in the differential responses of the KO clones compared to WT across experimental conditions (Fig. 5*D*, Table S4).

GSEA with mouse ortholog hallmark gene sets from molecular signatures database (MSigDB) on shared DEGs between FOXS1 KO clones 1 and 2 in TGF-β1 + DMII stimulated conditions revealed several hallmark pathways that were upregulated in the FOXS1 KO clones relative to WT 10T1/2 cells, including adipogenesis, UV response, p53 pathway, tumor necrosis factor alpha (TNFA) signaling *via* NFKB, bile acid metabolism, estrogen response late, and inflammatory response (Fig. 5*E*, Table S5). Adipogenesis hallmark pathway enrichment was driven by core genes that orchestrate adipogenesis and adipocyte functions, such as adipogenic TFs (*Pparg* and *Stat5a*), lipid metabolism, (*Acadm*, *Acox1*, *Cd36*, *Dgat1*, *Ephx2*, *Fabp4*, *Lipe*, *Ltc4s*, and *Plin2)*, and insulin sensitivity (*Adipoq*, *Adipor2*, *Enpp2*, and *Tst*) (Fig. 5*F*).

TGF-β1 downregulates many proadipogenic factors during myofibroblast activation, including PPARγ (30). We validated the upregulated expression of *Pparg* in TGF-β1 + DMII stimulated FOXS1 KO 10T1/2 cells relative to WT by RT-qPCR. *Pparg* was robustly DN in WT 10T1/2 cells but remained at significantly increased levels in each FOXS1 KO clone, despite TGF-β1 stimulation (Fig. 5*H*). Strikingly, *Cebpa*, *Cebpb*, and *Cebpd* were all significantly increased in each FOXS1 KO clone relative to WT after TGF-β1 + DMII stimulation (Fig. 5*H*). These data indicate that ablation of FOXS1 promotes proadipogenic gene signatures and abrogates TGF-β1-dependent regulation of crucial mediators of adipogenesis.

### Ablation of endogenous FOXS1 dampens molecular phenotypes associated with TGF-β1-dependent myofibroblast activation

Several GSEA hallmark pathways were DN in FOXS1 KO clones relative to WT 10T1/2 cells after TGF-β1 + DMII stimulation, including myc targets v1, myc target v2, apical junction, myogenesis, epithelial mesenchymal transition (EMT), hedgehog signaling, and KRAS signaling DN (Fig. 5*E*, Table S5). Although 10T1/2 cells are already mesenchymal, EMT pathways share considerable overlap with pathways and cellular processes involved in myofibroblast activation (31). EMT hallmark pathway enrichment was driven by a subset of genes that were DN in FOXS1 KO clones relative to WT (Fig. 5*G*). This subset included genes associated with cytoskeletal dynamics (*Acta2*, *Capg*, *Pdlim4*, *Tagln*, *Tpm1*, and *Tpm2*), cell adhesion (*Cdh6*, *Itga2*, and *Lrrc15*), and ECM remodeling (*Fbln1*, *Fbn1*, *Lama1*, *Lama2*, *Loxl1*, and *Postn*) (Fig. 5*G*). Many of these genes and cellular processes are crucial for profibrotic fibroblast activation, further implicating FOXS1 as a transcriptional regulator of TGF-β1-dependent myofibroblast activation.

We validated decreased upregulation of myofibroblast contractile markers (*Acta2* and *Tagln*) in the FOXS1 KO clones, along with profibrotic ECM markers (*Col1a1* and *Col3a1*), after TGF-β1 + DMII stimulation. Strikingly, FOXS1 KO clones had a significantly abrogated ability to upregulated *Acta2* and *Tagln* after TGF-β1 + DMII (Fig. 5*I*). However, FOXS1 KO 10T1/2 cells still significantly upregulated Col1a1 and Col3a1, with induction comparable to WT 10T1/2 cells (Fig. 5*I*).

## Discussion

We characterized FOXS1 as a regulator of TGF-β1-dependent profibrotic and antiadipogenic molecular phenotypes. We identified several profibrotic and adipogenic genes that are, in part, regulated by FOXS1 and determined that FOXS1 negatively regulates early adipogenic gene programs, which mitigates cellular adipogenic potential. Furthermore, FOXS1 sensitized 10T1/2 cells to TGF-β1 stimulation, potentiating TGF-β1-dependent blockade of terminal adipogenesis. FOXS1 KO 10T1/2 cells had a significantly higher adipogenic potential than their WT counterpart and maintained molecular phenotypes favoring adipogenesis and mitigating myofibroblast activation, despite TGF-β1 stimulation.

Previous studies have characterized FOXS1 as a profibrotic TGF-β-responsive TF in the liver and renal fibrosis (32, 33). FOXS1-dependent regulation of myofibroblast *Acta2* expression has been previously noted in hepatic stellate cells and dermal fibroblasts (32, 34). Hepatic stellate cell myofibroblast activation significantly contributes to liver fibrosis (35), further highlighting that FOXS1 may be crucial for aberrant myofibroblast activation in tissue-resident stem cells that drives tissue fibrosis.

Our results expand upon the role of FOXS1 as a determinant of TGF-β1-dependent myofibroblast activation. Notably, FOXS1 potentiated TGF-β1-dependent regulation of *Acta2*, *Tagln*, *Col1a1*, *Fn1*, ACLP/*Aebp1*, and *Il11* (Fig. 3*E*), which are established markers of fibrotic WAT remodeling and myofibroblast activation (11, 22, 36). Interestingly, ablating FOXS1 robustly abrogated TGF-β1-dependent upregulation of *Acta2* and *Tagln*, but not *Col1a1* or *Col3a1* (Fig. 5*J*), suggesting that FOXS1 may be a selective regulator of myofibroblast contractile phenotypes. Notably, ⍺-SMA/*Acta2* expression is sufficient to significantly abrogate mesenchymal stem cell adipogenesis (13). Although the role of ⍺-SMA^+^ myofibroblasts in WAT fibrosis is not completely understood, studies have shown the presence of ⍺-SMA^+^ ASC in WAT isolated from obese individuals, as well as increased WAT *Acta2* expression in mouse models of diet-induced obesity (12, 37). Differentiated FOXS1 OE 10T1/2 cells had significantly greater *Acta2* expression at D6 of adipogenesis compared to WT with TGF-β1 stimulation (Fig. 4*F*). Thus, FOXS1-dependent regulation of ⍺-SMA expression may contribute to the overall antiadipogenic properties of FOXS1 in this study. However, follow-up is needed to fully characterize the relationship between FOXS1 and ⍺-SMA regulation within the context of myofibroblast activation and adipogenic capacity.

In addition, FOXS1 is an established regulator of EMT in renal fibrosis and various cancers (33, 38, 39, 40). Although our cell-based assays relied on multipotent 10T1/2 fibroblasts that already have phenotypic features of mesenchymal cells, FOXS1 KO 10T1/2 clones had a DN EMT hallmark pathway signature that was driven by downregulation of several markers of activated myofibroblasts, including many involved in actin cytoskeletal regulation (Fig. 5*G*). Reorganization of the actin cytoskeleton is a crucial step during adipogenesis that allows for efficient fatty acid uptake and lipid droplet formation (41, 42). To that end, FOXS1-dependent regulation of cytoskeletal elements may explain why FOXS1 negatively regulates the expression of several lipid metabolism genes (Fig. 4*D*) and why a proadipogenic molecular signature, driven in part, by upregulation of several lipid mobilizing factors, was enriched in FOXS1 KO 10T1/2 cells (Fig. 5*F*). In addition to changes in cytoskeletal dynamics, adipogenic differentiation is dependent on cell cycle regulatory circuits as the cells exit the cell cycle and terminally differentiate (43). A potential involvement of FOXS1 in these cell cycle regulatory pathways in adipogenesis is supported by our finding that several cyclin dependent kinase inhibitors were differentially regulated in FOXS1 KO 10T1/2 cells compared with controls (Table S4).

Several FOX TFs have previously been described as negative regulators of adipogenesis, including FOXA2 (44), FOXC2 (45), FOXO1 (46), and FOXO3 (47). Of note, FOXC2 is closely related to FOXS1 in the FOX TF superfamily (48). FOXC2 represses adipogenesis by attenuating the transcriptional activity of PPARγ. (45). FOXC2 is also involved in matrix stiffness-dependent upregulation of the myofibroblast markers ⍺-SMA, COL1A1, and FN1 in hepatic stellate cells (49). Similarity between FOXC2-dependent and FOXS1-dependent molecular and cellular phenotypes suggests possible redundant regulatory functions between FOXC2 and FOXS1, which is a common property of FOX TFs due to their highly conserved winged-helix DNA-binding domain (48). However, FOXC2 has been shown to bind SMAD3 and SMAD4 to potentiate SMAD3/4 transcriptional activity (50). In our study, FOXS1 did not potentiate SMAD3/4 activation of *Acta2* or *Tagln* promoters (Fig. 3*F*), suggesting a possible mechanistic divergence between FOXC2 and FOXS1 functions. These divergent mechanisms may be explained, in part, by differences in the N- and C-terminal transactivation domains of FOXC2 and FOXS1. Compared to FOXC2, FOXS1 is not a significant transcriptional activator or repressor of promoters with FOX TF binding elements, further suggesting divergent molecular mechanisms for regulating cellular TGF-β1-responsiveness (51).

One study has noted that male FOXS1^β-gal/β-gal^ mice, which lack endogenous global FOXS1 tissue expression, had reduced weight gain with high-fat diet compared to littermate controls (52). More sophisticated mouse diet studies are needed to understand if this is due to WAT specific FOXS1 deletion and would allow for in-depth assessment of FOXS1-dependent fibrotic WAT remodeling in diet-induced obesity. Our studies revealed expression of FOXS1 RNA in the stromal vascular niche in close association with ⍺-SMA positive cells (Fig. 2*E*). These findings are consistent with single-cell data sets from mice fed a high-fat diet which shows expression of FOXS1 in adipogenic progenitor population (53). Additional studies are needed to fully define the expression dynamics and role of FOXS1 in adipose tissue remodeling.

In all, FOXS1 is a TGF-β1 downstream effector that promotes myofibroblast activation and mitigates fibroblast adipogenesis. These processes are critical in obesity-induced WAT fibrotic WAT remodeling, suggesting that FOXS1 may mediate TGF-β1-dependent profibrotic cellular responses and promote adipocyte hypertrophy in dysfunctional WAT. Further work is needed to underscore FOXS1 involvement in WAT fibrosis and dysfunction.

## Experimental procedures

### Mice

C57BL/6 mice were purchased from The Jackson Laboratory and housed in a temperature-controlled environment with a 12 h light-dark cycle, *ad libitum* water, and a standard chow diet. All animal studies were approved by the Boston University Chobanian & Avedisian School of Medicine Institutional Animal Care and Use Committee.

### Mouse WAT fractionation

WAT was fractionated into adipocytes and an SVF as previously described (22). In brief, WAT depots were washed three times in PBS and finely minced until no large tissue pieces were visible. Minced fat was digested in serum-free Dulbecco's modified Eagle's medium (DMEM) supplemented with 1% penicillin-streptomycin-glutamine (PSG) (Thermo Fisher Scientific), 1% bovine serum albumin (BSA) (Thermo Fisher Scientific), and 1 mg/ml type I collagenase (Worthington) for 45 min at 37 °C on a nutator. The digestion was quenched with DMEM supplemented with 10% fetal bovine serum (FBS) (Cytiva) and 1% PSG. The cell suspension was filtered through a 100 μm cell strainer and the flow-through was centrifuged at 300*g* for 10 min.

### Cell culture

10T1/2 cells (CCL-226, American Type Culture Collection [ATCC]) and HEK293T (CRL-3216, ATCC) cells were maintained in DMEM supplemented with 10% FBS (Cytiva) and 1% PSG. Deidentified primary omental hASC were obtained from the Boston Nutrition Obesity Research Center (BNORC). hASCs were maintained in modified Eagle's medium-⍺ supplemented with 10% FBS and 1% PSG. For 10T1/2 adipogenic differentiation, cells were grown to 2 days post confluence (>90%). Cells were then induced with 1:1 DMEM/Ham’s F-12 supplemented with 5 μM dexamethasone (Sigma-Aldrich), 500 μM 3-isobutyl-1-methylxanthine (Sigma-Aldrich), 860 nM insulin (Sigma-Aldrich), and 125 μM indomethacin (Sigma-Aldrich) for 2 days. Subsequently, cells were cultured in maintenance media (1:1 DMEM/Ham’s F-12 supplemented with 860 nM insulin) until the desired day of differentiation was reached. For TGF-β1 stimulations, 10T1/2 cells and hASC were serum-starved overnight and treated with 1 nM TGF-β1 (R&D) for the indicated time points. To inhibit TGF-β1-dependent SMAD2/3 phosphorylation, 10T1/2 cells were cotreated with 5 μM SB431542 (R&D) and 1 nM TGF-β1. For experiments that included TGF-β1 stimulation and adipogenic differentiation, 10T1/2 cells were treated with 1 nM TGF-β1 for the indicated time prior to adipogenic induction.

### Lentivirus and transduction

HEK293T cells were cotransfected using Lipofectamine 2000 with the lenti-packaging vectors Pax2 (#35002, Addgene) and pMD2.g (#12259, Addgene), along with transfer plasmid pLVX-IRES-Hyg FOXS1-HA. FOXS1-HA complementary DNA (cDNA) was cloned from a gBlock (IDT). Cell supernatant was collected 48 h post transfection, cleared by centrifugation at 1000*g* for 5 min, and passed through a 0.45 μm syringe filter. Virus titer was measured using a Lenti-X GoStix Plus (Takara). 10T1/2 cells were transduced by incubating cells with a mixture of cell culture media, lentivirus stock, polybrene (4 μg/ml), and Hepes (20 μM) for 24 h. Antibiotic selection began 48 h post transduction. 10T1/2 cells transduced with pLVX-IRES-Hyg FOXS1-HA lentivirus were selected with 250 μg/ml hygromycin B (Invitrogen) and maintained in 150 μg/ml hygromycin B.

### Bulk RNA-seq and analysis

Library preparation and bulk RNA sequencing was conducted by Novogene using the Illumina NovaSeq PE150 platform with paired-end sequencing (150-bp read length) and a 20 million read depth. Clean reads were mapped to either the human reference genome (GRCh38/hg38) or mouse reference genome (GRCm38/mm10) using HISAT2 software (v2.1.0). Differential gene expression analysis was performed on raw counts using the R statistical package DESeq2 (v1.28.1). Adjusted *p*-values were calculated using a 5% false discovery rate (FDR = 0.05) and the Benjamini-Hochberg procedure. Genes with a more than two-fold increase or decrease and an adjusted *p* < 0.05 were considered significantly differentially expressed. Pathway enrichment analysis was performed using GSEA software (v4.3.2).

### RT-qPCR

Total RNA was purified using the GeneJET RNA Purification Kit (Thermo Fisher Scientific) or the RNeasy Plus Mini Kit (Qiagen). RNA was quantified on a NanoDrop One using A260/280. cDNA was synthesized with the LunaScript RT SuperMix Kit (NEB) with 100 to 500 ng of total RNA. All cDNA was diluted 1:5 using nuclease-free water. qPCR was performed using Luna qPCR Master Mix (NEB) and a CFX Opus Real-Time PCR System (Bio-Rad). Relative gene expression (fold change) was calculated using the ΔΔCT method. CT values were normalized using 18s rRNA expression or *PPIA/Ppia* gene expression. See Table S6 for RT-qPCR primer sequences.

### CRISPR-Cas9 genome editing

Two pairs of guide RNAs (gRNAs) targeting the murine genomic FOXS1 exon were designed using CRISPOR software (v5.01). gRNA pairs were annealed and ligated into the PX459 vector (#62988, Addgene). 10T1/2 cells were cotransfected with both FOXS1 gRNA constructs and selected with puromycin (1 μg/ml) for 48 h. Single-cell colonies were grown in 10T1/2 conditioned culture media and expanded in growth media. Disruption of the FOXS1 locus was determined by genomic DNA sequencing and loss of FOXS1 expression was confirmed by western blotting. See Table S7 for FOXS1 gRNAs.

### Western blotting

Cultured cell pellets were lysed on ice in lysis buffer (20 mM Tris pH 7.5, 150 mM NaCl, 1 mM Na_2_EDTA, 1 mM EGTA, and 1% Triton-X 100) supplemented with protease and phosphatase inhibitors (Roche). Cell lysates were cleared by centrifugation at 17,000*g* for 15 min (4 °C). Total protein concentration was determined using the Pierce BCA Protein Quantification Kit (Thermo Fisher Scientific). Protein samples were boiled at 95 °C for 5 min and resolved on 12% Tris-glycine SDS-PAGE gels (Invitrogen). Samples were transferred overnight onto nitrocellulose. Membranes were blocked with 4% milk in tris-buffered saline with tween 20 (TBST) for 1 h at room temperature (RT) and probed with primary antibody in 4% milk TBST (2 h, RT). Blots were probed with horseradish peroxidase (HRP)-conjugated secondary antibodies in 4% milk TBST (1 h, RT). Chemiluminescent signal was detected using SuperSignal West Femto Substrate (Thermo Fisher Scientific) or SuperSignal West Dura Substrate (Thermo Fisher Scientific). Blots were imaged using a ChemiDoc Imaging System (Bio-Rad). The following antibodies were used for western blot detection: FOXS1 (1:600; #16234-1-AP, Proteintech), anti-HA Tag (1:1000; #13-2010, EpiCypher), phospho-SMAD2/3 (1:1000; #8828, Cell Signaling), total SMAD2/3 (1:1000; #8685, Cell Signaling), GAPDH (1:1000; #97166, Cell Signaling), Cyclophilin A (1:1000; #51418, Cell Signaling), mouse IgG HRP secondary (1:4000; #NA931, Cytiva), and rabbit IgG HRP secondary (1:4000; #NA934, Cytiva). Specificity was determined using several approaches including the appropriate molecular weight or subcellular localization for immunofluorescence studies, verification from other studies in the literature, and by detection in overexpressed or loss in KO cells.

### Luciferase reporter assays

10T1/2 cells were cotransfected with pGL4.10-*Acta2* or pGL4.10-*Tagln* promoter luciferase reporter vectors, along with CMV-5β-FOXS1, CMV-5⍺-SRF, pCL-SMAD3, and CMV-5β-SMAD4 expression vectors, using TransIT-X2 (Mirus Bio) transfection reagent. Cotransfection with CMV5β-β-galactosidase was used as an internal control. Using the Luciferase Assay System (Promega), cell lysates were collected and frozen at −80 °C for 24 h post transfection. Luciferase expression was measured using a BioTek Synergy HT plate reader and BioTek Gen5 software (v3.11). β-galactosidase expression was determined by incubating cell lysates with ortho-nitrophenyl-β-galactoside at 37 °C and reading the absorbance at 405 nm as described (23). Luciferase expression was normalized by dividing the relative light unit values by the β-galactosidase absorbance for each sample.

### Immunofluorescence

Cultured cells were fixed in 4% paraformaldehyde for 15 min at RT and washed two times with PBS. Fixed cells were permeabilized in PBS + 0.1% Triton-X 100 for 10 min at RT then washed twice with PBS. Cells were blocked for 1 h in PBS + 5% BSA. Cells incubated with primary antibody diluted in PBS + 1% BSA overnight at 4 °C. Afterward, cells were washed five times with PBS and incubated with secondary antibody diluted in PBS + 1% BSA for 1 h in the dark. Cells were washed five times with PBS and stained with Hoechst nuclear stain (Invitrogen, 1:1000). Images were taken using an EVOS M5000 imaging system (Thermo Fisher Scientific). Fluorescent intensity was measured using background corrected images in Fiji (v1.54p).

### RNAscope

Mouse iWAT and gWAT tissue was processed as described (22). Deparaffinized tissue sections were subjected to antigen recovery using 1X ACD RNAscope target retrieval buffer at 110 °C for 15 min (pressure cooker). RNAscope was performed using the RNAscope Multiplex Fluorescent Reagent Kit v2 (ACD) with a Mm-Foxs1-C2 (ACD, 1232131-C2; 1:50) probe and TSA vivid dye 650 (ACD, 1:1000). Tissues were co-stained with ⍺-SMA (Sigma-Aldrich, C6198; 1:300) and mounted with prolong diamond antifade mountant with 4′,6-diamidino-2-phenylindole (DAPI) (Thermo Fisher Scientific). Tissues were imaged using a Zeiss Observer D1 equipped with an ORCA-Flash 4.0 digital CMOS camera with equivalent exposure time.

### Oil Red-O staining

Differentiated cells were fixed in 4% paraformaldehyde for 15 min at RT and washed twice with PBS. Cells were washed with 60% isopropanol for 5 min at RT. Oil Red-O stock was diluted 3:2 with water and filtered through a 0.22 μm syringe filter. Cells were stained with Oil Red-O for 5 min at RT and washed four times with water. Cells were kept in PBS for imaging using an EVOS M5000 imaging system (Thermo Fisher Scientific). To quantify Oil Red-O staining, cells were incubated with 100% isopropanol for 10 min at RT to strip the stain. The absorbance of the isopropanol was read at 500 nm using a BioTek Synergy HT plate reader and Gen5 software (v3.11). Empty wells stained with Oil Red-O were used as a blank control as described (22).

### Statistical analysis

Data are presented as mean ± SD. Statistical significance was determined by one-way or two-way analysis of variance (ANOVA) with Bonferroni correction for multiple comparisons when comparing more than two experimental groups. Statistical significance was defined as *p* < 0.05.

## Data availability

The RNA sequencing data that support the findings of this study are publicly available in the Gene Expression Omnibus (GEO) repository under the accession numbers: GSE302340, GSE302341.

## Supporting information

This article contains supporting information.

## Conflict of interest

The authors declare that they have no conflicts of interest with the contents of this article.
