# Supplementary material for: TGF-β1-dependent expression of FOXS1 attenuates adipogenic potential and enhances a myofibroblast cellular phenotype
Source: J Biol Chem. 2025 Aug 5;301(9):110563. doi: 10.1016/j.jbc.2025.110563 (PMC12423405; doi:10.1016/j.jbc.2025.110563)
Supplement: Supporting Figure S1 [file mmc1.pdf]

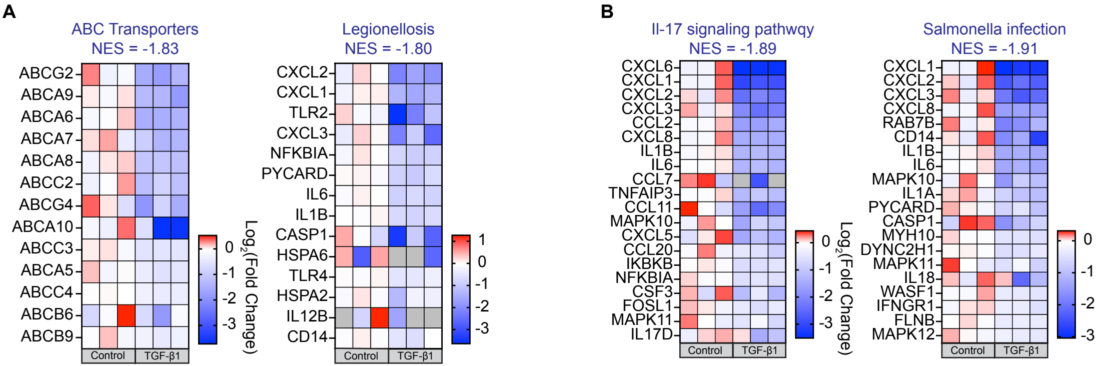


**Figure S1. GSEA downregulated KEGG pathways in 12 h and 72 h TGF-β1 stimulated hASC. (A)** Core gene enrichments for downregulated ABC transporters and legionellosis KEGG pathways in 12 h TGF-β1 stimulated hASC. **(B)** Core gene enrichments for downregulated Il-17 signaling and salmonella infection KEGG pathways in 72 h TGF-β1 stimulated hASC. NES = normalized enrichment score. Gene expression is reported as log2(fold change) relative to time-matched controls.
