# Supplementary material for: TGF-β1-dependent expression of FOXS1 attenuates adipogenic potential and enhances a myofibroblast cellular phenotype
Source: J Biol Chem. 2025 Aug 5;301(9):110563. doi: 10.1016/j.jbc.2025.110563 (PMC12423405; doi:10.1016/j.jbc.2025.110563)
Supplement: Supporting Figure S2 [file mmc2.pdf]

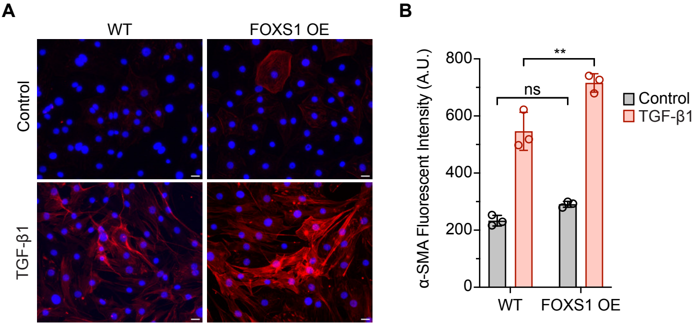

Figure S2. FOXS1 potentiates the TGF-β1-dependent upregulation of α-SMA. (A) Wildtype (WT) and FOXS1 OE 10T1/2 cells stimulated with TGF-β1 (1 nM) for 48 hours. Representative images (n = 3) were stained for α-SMA (red) and Hoescht (blue). **(B)** Quantification of the α-SMA fluorescent intensity. Two-way ANOVA with Bonferroni corrections for multiple comparisons (ns = not significant, ** $P < 0.01$). Data presented as mean ± S.D. Scale bars = 25 μm.
