# Supplementary material for: TGF-β1-dependent expression of FOXS1 attenuates adipogenic potential and enhances a myofibroblast cellular phenotype
Source: J Biol Chem. 2025 Aug 5;301(9):110563. doi: 10.1016/j.jbc.2025.110563 (PMC12423405; doi:10.1016/j.jbc.2025.110563)
Supplement: Supporting Tables S6 and S7 [file mmc8.pdf]

Table S6. RT-qPCR Primers

Gene	Forward 5’ → 3’	Reverse 5’ → 3’	Species
Ppia	GAGCTGTTTGCAGACAAAGTTC	CCCTGGCACATGAATCCTGG	Mouse
18s	CGGCTACCACATCCAAGGAA	TTTTCGTCACTACCTCCCCG	Mouse
Foxs1	ACAGCTACATAGCCCTGATTGC	AGAGACAGGTTATGGCGGATG	Mouse
Acta2	GTCCCAGACATCAGGGAGTAA	TCGGATACTTCAGCGTCAGGA	Mouse
Tagln	CAACAAGGGTCCATCCTACGG	ATCTGGGCGGCCTACATCA	Mouse
Col1a1	GCTCCTCTTAGGGGCCACT	CCACGTCTCACCATTGGGG	Mouse
Col3a1	CTGTAACATGGAAACTGGGGAAA	CCATAGCTGAACTGAAAACCACC	Mouse
Fn1	ATGTGGACCCCTCCTGATAGT	GCCCAGTGATTTCAGCAAAGG	Mouse
Aebp1	ATAGCTACGTGATCCCCAAC	GTGGGCACTTGATTTTCTCC	Mouse
Il11	TGTTCTCCTAACCCGATCCCT	CAGGAAGCTGCAAAGATCCCA	Mouse
Pparg	GGAAGACCACTCGCATTCCTT	GTAATCAGCAACCATTGGGTCA	Mouse
Cebpa	CAAGAACAGCAACGAGTACCG	GTCACTGGTCAACTCCAGCAC	Mouse
Cebpb	CAACCTGGAGACGCAGCACAAG	GCTTGAACAAGTTCCGCAGGGT	Mouse
Cebpd	CGACTTCAGCGCCTACATTGA	CTAGCGACAGACCCCACAC	Mouse
Stat5a	CGCCAGATGCAAGTGTTGTAT	TCCTGGGGATTATCCAAGTCAAT	Mouse
Adipoq	TGTTCCTCTTAATCCTGCCCA	CCAACCTGCACAAGTTCCCTT	Mouse
Fabp4	AAGGTGAAGAGCATCATAACCCT	TCACGCCTTTCATAACACATTCC	Mouse
Plin1	GGGACCTGTGAGTGCTTCC	GTATTGAAGAGCCGGGATCTTTT	Mouse
Plin2	GACCTTGTGTCCTCCGCTTAT	CAACCGCAATTTGTGGCTC	Mouse
18S	CGGCGACGACCCATTCGAAC	GAATCGAACCCTGATTCCCCGTC	Human
FOXS1	AGTGGCATCTACCGCTACATC	CACCTTGACAAAGCACTCGT	Human

Table S7. FOXS1 gRNAs

	Sense gRNA	Antisense gRNA
FOXS1 gRNA #1	CGGCCGGTTGTGCCGGTAAA	TTTACCGGCACAACCGGCCG
FOXS1 gRNA #2	TGTCGTGGCAGTCCGGATCC	GGATCCGGACTGCCACGACA
